# Schizophrenia and type 2 diabetes risk: a systematic review and meta-analysis

**DOI:** 10.3389/fendo.2024.1395771

**Published:** 2024-09-11

**Authors:** Kai Dong, Shenghai Wang, Chunhui Qu, Kewei Zheng, Ping Sun

**Affiliations:** ^1^ College of Mental Health, Jining Medical University, Jining, China; ^2^ Qingdao Mental Health Center, Qingdao, China; ^3^ College of Special Education and Rehabilitation, Binzhou Medical University, Yantai, China

**Keywords:** schizophrenia, type 2 diabetes mellitus, T2DM, systematic review, meta-analysis, observational study

## Abstract

**Objectives:**

The metabolic syndrome in patients with schizophrenia has consistently been a challenge for clinicians. Previous studies indicate that individuals with schizophrenia are highly prone to developing type 2 diabetes mellitus (T2DM). In recent years, a continuous stream of new observational studies has been reported, emphasizing the pressing need for clinicians to gain a more precise understanding of the association between schizophrenia and T2DM. The objective of this meta-analysis is to integrate new observational studies and further explore the potential link between schizophrenia and the risk of T2DM.

**Methods:**

We conducted a comprehensive search of PubMed, Cochrane Library, Embase, and Web of Science using medical subject headings (MeSH) and relevant keywords. The risk of bias in cohort studies and case-control studies was assessed using the Newcastle-Ottawa Scale (NOS), while cross-sectional studies were evaluated using the Agency for Healthcare Research and Quality scale (AHRQ), scoring was based on the content of the original studies. A fixed-effects model was employed if P > 0.1 and I2 ≤ 50%, indicating low heterogeneity. Conversely, a random-effects model was utilized if I2 > 50%, indicating substantial heterogeneity. Publication bias was assessed using funnel plots and Egger’s test. Statistical analyses were carried out using Stata statistical software version 14.0.

**Results:**

This meta-analysis comprised 32 observational studies, involving a total of 2,007,168 patients with schizophrenia and 35,883,980 without schizophrenia, published from 2004 to 2023. The pooled analysis revealed a significant association between a history of schizophrenia and an increased risk of T2DM (Odds Ratio [OR] = 2.15; 95% Confidence Interval [CI]: 1.83–2.52; I2 = 98.9%, P < 0.001). Stratified by gender, females with schizophrenia (OR = 2.12; 95% CI: 1.70-2.64; I2 = 90.7%, P < 0.001) had a significantly higher risk of T2DM than males (OR = 1.68; 95% CI: 1.39-2.04; I2 = 91.3%, P < 0.001). Regarding WHO regions, EURO (OR = 2.73; 95% CI: 2.23-3.35; I2 = 97.5%, P < 0.001) exhibited a significantly higher risk of T2DM compared to WPRO (OR = 1.72; 95% CI: 1.32-2.23; I2 = 95.2%, P < 0.001) and AMRO (OR = 1.82; 95% CI: 1.40-2.37; I2 = 99.1%, P < 0.001). In terms of follow-up years, the >20 years subgroup (OR = 3.17; 95% CI: 1.24-8.11; I2 = 99.4%, P < 0.001) showed a significantly higher risk of T2DM than the 10-20 years group (OR = 2.26; 95% CI: 1.76-2.90; I2 = 98.6%, P < 0.001) and <10 years group (OR = 1.68; 95% CI: 1.30-2.19; I2 = 95.4%, P < 0.001).

**Conclusions:**

This meta-analysis indicates a strong association between schizophrenia and an elevated risk of developing diabetes, suggesting that schizophrenia may function as an independent risk factor for T2DM.

**Systematic review registration:**

https://www.crd.york.ac.uk/PROSPERO/, identifier CRD42023465826.

## 1 Backgrounds

Schizophrenia stands as a severe and debilitating mental illness characterized by its high prevalence, significant disability rate, and considerable overall disease burden (1). Individuals grappling with schizophrenia face a dramatically elevated all-cause mortality rate when compared to those without the condition, resulting in a substantial life expectancy gap of approximately 15 to 20 years (2, 3). In addition to factors such as suicide, accidents, and risky behaviors, cardiovascular disease emerges as a major contributor to the premature death often seen in individuals with schizophrenia (4, 5). Among the various risk factors contributing to cardiovascular disease, metabolic syndrome is an unavoidable topic, with T2DM being a significant component (6). On a global scale, T2DM represents a major health challenge. As of 2021, estimates indicate that around 537 million individuals worldwide grapple with T2DM, with a projected increase of 46% anticipated to reach 783 million by 2045 (7).

Prior investigations indicate that individuals with schizophrenia exhibit more severe blood sugar levels and insulin status than their healthy counterparts (8–11). Previous studies have attempted to explain the above phenomenon from different perspectives. From a genetic perspective, schizophrenia and T2DM have a significant genetic correlation (12), one compelling piece of evidence is the transcription factor 7-like 2 (TCF7L2) gene, which is identified as one of the most significant risk genes for T2DM (13), also has a significant contribution to schizophrenia (14). In terms of lifestyle habits, sedentary behavior and poor dietary habits are considered traditional factors leading to diabetes in patients with schizophrenia (15). For the treatment of schizophrenia, antipsychotics (AP), particularly second-generation antipsychotics (SGAs), are a standard approach, but while improving psychotic symptoms, they significantly impact metabolic levels, leading to T2DM (16–20), and studies on gut microbiota (GMB) have found that these medications alter GMB distribution, disrupt glucose tolerance, and exacerbate the trend of comorbid schizophrenia and T2DM (21), beyond the effects of medication, schizophrenia and T2DM themselves share a high degree similarities in GBM (22). The protracted course of T2DM can lead to complications such as cardiovascular disease and chronic kidney disease (23), and when combined with schizophrenia, it results in greater cognitive impairment (24), which contributes to a more severe prognosis for these individuals (25, 26). Notably, the Canadian Diabetes Association has identified schizophrenia as a risk factor for T2DM (27).

Despite extensive investigations into the various mechanisms linking schizophrenia and T2DM, a conclusive understanding remains elusive. While previous meta-analyses have reinforced the association between schizophrenia and T2DM (28, 29), they have not delved into additional subgroup analyses, such as those stratified by gender, WHO region, study type, or study period. Concurrently, a multitude of new observational studies has emerged. Consequently, we undertook a thorough review of these recent observational studies and existing meta-analyses to elucidate pertinent findings and offer the most up-to-date evidence on the correlation between schizophrenia and T2DM. Our objective is to enable clinicians to promptly refine treatment strategies, thereby enhancing the quality of life and extending the life expectancy of individuals contending with schizophrenia.

## 2 Methods

This meta-analysis adhered to the guidelines outlined in the Preferred Reporting Items for Systematic Reviews and Meta-Analyses (PRISMA) (30). The research protocol was pre-registered on the International Prospective Register of Systematic Reviews (PROSPERO) platform, with the approval number CRD42023465826.

### 2.1 Data sources and searches

We conducted searches on PubMed, Cochrane Library, Embase, and Web of Science to identify observational studies published from the inception of the databases to September 19, 2023. The language was restricted to English, and our search strategy incorporated a combination of medical subject headings (MeSH) and keywords. The search terms encompassed a range of topics, including schizophrenia, schizophreni*, Dementia Praecox, Diabetes Mellitus, Diabetes Insipidus, Diet, Diabetic, Prediabetic State, Scleredema Adultorum, Glucose Intolerance, and Gastroparesis. Additionally, we scrutinized the reference lists of included cohort studies, case-control studies, cross-sectional studies, and other published meta-analyses to identify relevant trials.

### 2.2 Eligibility criteria

The inclusion criteria for trials were as follows (1): observational studies were considered, with the exception of intervention studies (2); the observation group comprised patients diagnosed with schizophrenia, while the control group consisted of individuals without schizophrenia or comparisons were made with large datasets containing prevalence data on T2DM (3); the original study should accurately diagnose both schizophrenia and T2DM (4); trials that did not recruit a control group but utilized previously published general population data were considered (5); preference was given to trials that included both baseline and follow-up data, with prioritization given to the latter. Trials with low NOS or AHRQ scores were excluded. In cases where multiple studies reported data from the same cohort, priority was given to the study with the longest follow-up or the largest number of participants. Trials presenting excessively wide 95% confidence intervals (CI) were excluded. Additionally, the following types of articles were excluded: conference abstracts, study protocols, duplicate publications, and studies lacking outcomes of interest. In instances of mixed samples, efforts were made to extract data specifically related to individuals with schizophrenia. If such data extraction was not feasible, attempts were made to contact the authors up to two times within a one-month period to obtain data specifically for individuals with schizophrenia. Trials where contact was unsuccessful were excluded.

### 2.3 Study selection

Two reviewers (KD and PS) independently screened the literature based on the eligibility and exclusion criteria. Initially, duplicate and irrelevant articles were excluded by assessing their titles and abstracts. Subsequently, the full texts of potentially eligible articles were retrieved and thoroughly reviewed to identify all suitable studies. Any discrepancies were resolved through discussion with a third reviewer (PS), serving as an arbiter.

### 2.4 Data extraction

The process of data extraction was meticulously carried out by the two aforementioned reviewers (DK, SHW,CHQ, KWZ), who referred to established guidelines for systematic reviews and meta-analysis (31). Utilizing predefined forms, they systematically extracted key information such as the first author, year of publication, country, WHO region, study type, sample size, follow-up years, year of data collection, percentage of males, age, diagnosis of schizophrenia/T2DM, and adjustments made for confounders. In instances where discrepancies arose, the reviewers engaged in thorough discussions with PS, serving as a mediator, to achieve a consensus and ensure the accuracy and reliability of the extracted data.

### 2.5 Risk of bias assessment

To gauge the methodological quality of cohort and case-control studies, the NOS was employed (32). The scoring system allocated stars on a scale of 0 to 9 for both cohort and case-control studies, with four stars designated for the selection of participants and measurement of exposure, two stars for comparability, and three stars for the assessment of outcomes and adequacy of follow-up. A higher number of stars signified a higher quality of the study. Scores falling within the ranges of 0–3, 4–6, and 7–9 were categorized as indicating low, moderate, and high quality, respectively. For the evaluation of cross-sectional studies, the AHRQ was employed (33). This scale comprises 11 items, with each item assessed using “yes”, “no”, or “unclear”. The scoring method involves assigning points for each “yes” response, resulting in a total score ranging from 0 to 11 points. Scores within the ranges of 0–3, 4–7, and 8–11 were interpreted as indicative of low, moderate, and high quality, respectively.

### 2.6 Statistical analysis

To assess the association between schizophrenia and the risk of diabetes, the adjusted odds ratios (OR) and their corresponding 95% confidence intervals (CI) from each trial were utilized. Heterogeneity was evaluated using the χ2-test and I2-values. In cases where P > 0.1 and I2 ≤ 50%, indicating minimal heterogeneity, a fixed-effects model was employed. However, if I2 > 50%, suggesting significant heterogeneity, a random-effects model was applied. To ensure the robustness of the overall effects, a sensitivity analysis was conducted by systematically excluding one study at a time and re-running the analysis. Publication bias was visually inspected through a funnel plot, and Egger’s regression test was employed for a statistical assessment of publication bias. Subgroup analyses were performed based on gender, study type, WHO region, year of data collection, and follow-up time to provide a more nuanced understanding of the results. All statistical analyses were executed using Stata statistical software version 14.0 (Stata Corp, College Station, Texas).

## 3 Results

### 3.1 Literature search

**Figure 1:**
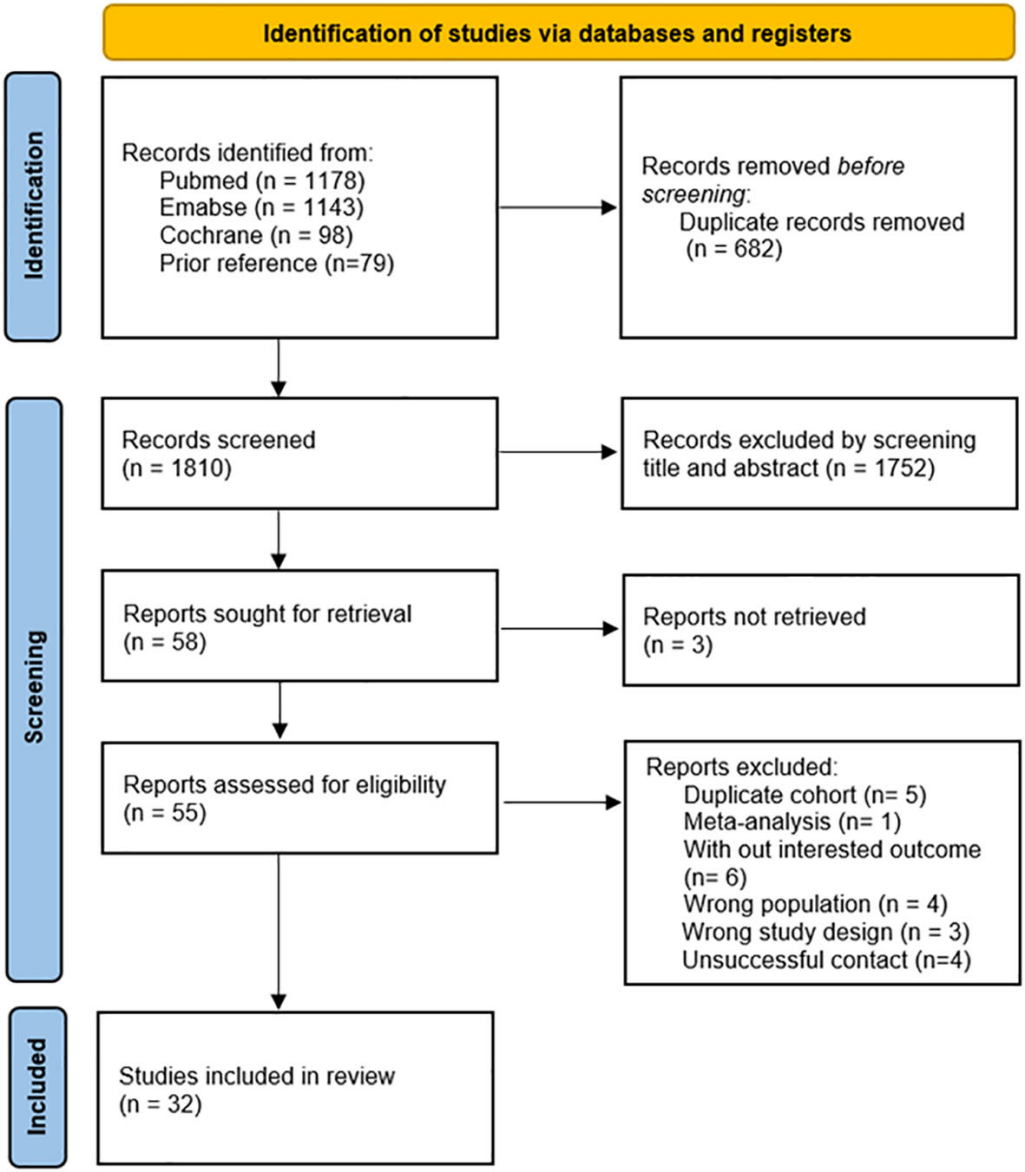
Studies screening process.

A systematic search of observational studies published up to September 19, 2023, generated a total of 2,419 results. Upon the removal of duplicate entries, the screening process involved the assessment of 1,810 abstracts and titles (**Figure 1**). Following this initial screening, 55 articles were identified as potentially relevant, of which 23 were subsequently excluded with detailed reasons provided. Ultimately, after a comprehensive full-text review, 32 studies (34–65) were included in the analysis. **Figure 1** provides a concise summary of the search results, elucidating the rationale behind the exclusion of specific articles.

### 3.2 Study characteristics

This meta-analysis aggregates findings from 32 observational studies, encompassing a substantial cohort of 2,007,168 individuals diagnosed with schizophrenia, alongside a comparison group comprising 35,883,980 individuals without schizophrenia. These studies were conducted and published between 2004 and 2023, showcasing a broad spectrum of research methodologies. Among them, 14 were cohort studies, two were case-control studies, and the remainder consisted of fifteen cross-sectional studies. The majority of participants in these investigations commenced follow-up at the age of 16 or older, with only one study focusing on individuals aged 0 to 36 years. Across all studies, diagnostic criteria for schizophrenia were consistently applied, ensuring a uniform standard across the analysis. The duration of follow-up varied across studies, ranging from 1 to 36 years, with one study exclusively focusing on a male cohort. Notably, adjusted estimates were available for nearly all studies, although adjustments for confounding variables may have differed slightly between studies. Detailed characteristics of the included trials are provided in **Table 1** for reference and clarity.

**Table 1:** Basic characteristics of the included studies.

Author	Year	Country	WHO region	Study type	Sample size and prevalence	Follow-up years or median	Year of data collection	Male, %	Age, mean, median or range	Diagnosis criteria	Confounders adjusted	Quality scores
Lee et al. (36)	2023	South Korea	WPRO	Cohort study	Schizophrenia: 313/7,408 No Schizophrenia: 122,290/6,450,583	7.59	2018	Total 59.2	Total 30.8	Schizophrenia: ICD-10 Diabetes: ICD-10	Age, gender, income, alcohol consumption, smoking status, physical activity, and metabolic syndrome	NOS scores 8
Shamsutdinova et al. (34)	2023	UK	EURO	Cross-sectional study	Schizophrenia: 1,160/7,392 No Schizophrenia: 36,862/666,885	NR	2013	Total 51.5	Total 38.0	Schizophrenia: ICD-9 Diabetes: ICD-9	NR	AHRQ scores 8
Matsunaga et al. (35)	2023	Japan	WPRO	Cross-sectional study	Schizophrenia: 23/223 No Schizophrenia: 56/1,776	NR	2022	Schizophrenia: 51.6 No schizophrenia: 45.1	Male: schizophrenia 48.0 No schizophrenia: 48.0 Female: Schizophrenia: 44.0 No schizophrenia: 42.0	Schizophrenia: Self-report (Based on the DSM-5) questionnaire Diabetes: Self-report	Age, gender	AHRQ scores 7
Lambert et al. (37)	2023	Australia	WPRO	Cross-sectional study	Schizophrenia: 212/888 No Schizophrenia: 110/514	5	2019	Total 63.1	Total 43.9	Schizophrenia: ICD-10 Diabetes: ADA	NR	AHRQ Scores 7
Gao et al. (38)	2022	USA	AMRO	Cohort study	Schizophrenia: 266, 012/1,785,314 No Schizophrenia: 2,602, 551/14,458,616	25	2018	Schizophrenia: 60.8 No Schizophrenia: 41.4	Schizophrenia: 43.9 No schizophrenia: 56.9	Schizophrenia: ICD-9, ICD-10 Diabetes: ICD-9, ICD-10	Age, year, and exposure main effects	NOS scores5
Melkersson et al. (40)	2020	Sweden	EURO	Cohort study	Schizophrenia: 18/1,465 No Schizophrenia: 2,002/1,734,816	median 10.6	2018	Schizophrenia: 68.2 No Schizophrenia: 51.4	Schizophrenia with T2DM:23.9 (median)	Schizophrenia: ICD-7, 8, 9, 10 Diabetes: ICD-7, 8, 9, 10	Gender, gestational age, birth weight in relation to gestational age, maternal smoking during pregnancy (only data from early pregnancy was available), parity, heredity for schizophrenia or schizoaffective disorder, and heredity for T1DM or T2DM.	NOS scores 8
Yang et al. (39)	2020	China	WPRO	Cohort study	Schizophrenia: 7,270/62,533 No Schizophrenia: 9,669/95,037	14 Averages	2018	Total 49.7	Total 43.5	Schizophrenia: ICD-10 Diabetes: ICD-10	Gender, age, ethnic origin, marital status, payment, and hospital level	NOS scores 7
Alonso et al. (41)	2020	Spain	EURO	Cross-sectional study	Schizophrenia: 12/164 No Schizophrenia: 14/156	NR	NR	Schizophrenia: 59.8 No Schizophrenia: 60.3	Schizophrenia: 42.8 Population control: 43.3	Schizophrenia: DSM-4 Diabetes: NR	NR	AHRQ scores 5
Pearsall et al. (42)	2019	UK(Scotland)	EURO	Cross-sectional study	Schizophrenia:364/3,154 No Schizophrenia: 207/2,696	NR	2015	Schizophrenia: 41.0 All samples: 56.1	>16	Schizophrenia: ICD-10 Diabetes: WHO	Age, sex, deprivation quintile and diagnosis	AHRQ scores 10
Jackson et al. (43)	2019	UK(Scotland)	EURO	Cohort study	Schizophrenia: 271/2,315 No Schizophrenia: 15, 320/246,046	15 Averages	2015	Schizophrenia: 58.8 No Schizophrenia: 55.6	Schizophrenia: 51.4 No Schizophrenia: 60.8	Schizophrenia: ICD-10 Diabetes: NR	Age	NOS scores 6
Garriga et al. (44)	2019	Denmark	EURO	Cohort study	Schizophrenia: 56/387 No Schizophrenia: 1,264/10,476	47.1 Averages	2018	100	18-65	Schizophrenia: ICD-8, ICD-10 Diabetes: ICD-8, ICD-10	Mother’s age, father’s occupational status, education, IQ, BMI at conscription,Birth Weight (and also includes adjustment for birth length), and Ponderal Index	NOS scores 8
Bent-Ennakhil et al. (46)	2018	Sweden	EURO	Cohort study	Schizophrenia: 219/2,530 No Schizophrenia: 6822/200,644	32 Averages	2012	Schizophrenia: 51.7 No Schizophrenia: 47.5	Schizophrenia: 42.7 No Schizophrenia: 43.8	Schizophrenia: ICD-10 Diabetes: ICD-10	Age, gender	NOS scores 8
Chiu et al. (45)	2018	Canada	AMRO	Cross-sectional study	Schizophrenia: 133/1,103 No Schizophrenia: 8914/156,376	NR	2010	Schizophrenia: 52.1 No Schizophrenia: 48.9	Schizophrenia: 46.4 No Schizophrenia: 44.6	Schizophrenia: CCHS survey data Diabetes: self-report	Age, gender	AHRQ scores 5
Brostedt et al. (50)	2017	Sweden	EURO	Cross-sectional study	Schizophrenia: 911/7,284 No Schizophrenia: 908/11,485	NR	2012	Schizophrenia: 57.0 No Schizophrenia: 49.0	Schizophrenia: 52.7 No Schizophrenia: 49.5	Schizophrenia: ICD-10 Diabetes: ICD-10	Age, gender	AHRQ scores 5
Jahrami et al. (48)	2017	Bahrain	EMRO	Case-control study	Schizophrenia: 37/120 No Schizophrenia: 12/120	NR	2016	Schizophrenia: 55.0 No Schizophrenia: 45.0	Schizophrenia: 41.7 No Schizophrenia: 41.7	Schizophrenia: ICD-10 Diabetes: ICD-10	Age, gender	NOS scores 7
Rajkumar et al. (47)	2017	Denmark	EURO	Cohort study	Schizophrenia: 25/1,154 No Schizophrenia:7,217/2,673,114	36	2013	Schizophrenia: 59.7 No Schizophrenia: 50.8	0-36	Schizophrenia: ICD-8, ICD-10 Diabetes: ICD-8, ICD-10	Gender, family history of diabetes, urbanicity, exposure to valproate, and exposure to tricyclic or tetracyclic antidepressants	NOS scores 8
Annamalai et al. (51)	2017	USA	AMRO	Cohort Study	Schizophrenia: NR/326 No Schizophrenia: NR/1,899	1	NR	Schizophrenia: 58.3 No Schizophrenia: 40.7	Schizophrenia: 47.5 No Schizophrenia: 55.1	Schizophrenia: DSM-4 Diabetes: Self-report	Age, sex(male), race(non-white), obesity and schizophrenia	NOS scores 7
Gabilondo et al. (49)	2017	Spain	EURO	Cross-sectional study	Schizophrenia: 845/7,331 No Schizophrenia: 139, 892/2,248,075	NR	2011	Schizophrenia: 57.69 No Schizophrenia: 49.04	Schizophrenia: 48.6 No Schizophrenia: 43.9	Schizophrenia: ICD-10 Diabetes: Barnett’s list and the ACG Technical Reference Guide	Age, sex and deprivation index	AHRQ scores 7
Schoepf et al. (52)	2014	UK	EURO	Case-control study	Schizophrenia: 247/1,418 No Schizophrenia: 1211/14,180	11.5	2012	Schizophrenia: 60.60 No Schizophrenia: 60.6	Schizophrenia: 49.8 No Schizophrenia: 50.1	Schizophrenia: ICD-10 Diabetes: ICD-10	Age, gender, ethnicity, time of follow-up duration (days), and the various comorbid diseases	NOS scores 7
Crump et al. (53)	2013	Sweden	EURO	Cohort Study	Schizophrenia: 963/8,277 No Schizophrenia: 348,736/6,089,577	7	2009	Schizophrenia: 57.8 No Schizophrenia: 48.7	>25	Schizophrenia: ICD-10 Diabetes: ICD-10	Age, Other Sociodemographic Variables (included marital status, education, employment status, and income), and Substance Use Disorders (included any outpatient or inpatient diagnosis of a substance use disorder)	NOS scores 7
Morden et al. (54)	2012	USA	AMRO	Cross-sectional study	Schizophrenia: 17,518/65,362 No Schizophrenia: 17,321/65,362	NR	2007	Schizophrenia: 87.9 No Schizophrenia: 87.9	Schizophrenia: 53.4 No Schizophrenia: 53.8	Schizophrenia: ICD-9 Diabetes: ICD-9	Age, gender, parent VA medical center and visit date	AHRQ scores 7
Mai et al (57)	2011	Australia	WPRO	Cohort Study	Schizophrenia: 109/818 No Schizophrenia: 1625/26,626	16	2006	Schizophrenia: NR No Schizophrenia: 47.8	>20	Schizophrenia: ICD-9 Diabetes: ICD-9	Five-year age group, sex, Indigenous status, level of social disadvantage, level of residential remoteness, physical comorbidities, calendar year and whether diabetes was identified before T0 and type of diabetic treatment	NOS scores 6
Zhang et al. (55)	2011	China	WPRO	Cross-sectional study	Schizophrenia: 46/206 No Schizophrenia: 38/615	NR	NR	NR	25~70	Schizophrenia: DSM IV Diabetes: WHO criteria	Age, gender, education, and body mass index (BMI)	AHRQ scores 4
Subashini et al (56)	2011	India	SEARO	Cross-sectional study	Schizophrenia: 20/131 No Schizophrenia: 38/524	NR	NR	Schizophrenia: 51.9 No Schizophrenia: 51.9	Schizophrenia: 44.0 No Schizophrenia: 44.0	Schizophrenia: DSM IV Diabetes: Self-report or ADA criteria	Age, sex	AHRQ scores 6
Hsu et al. (58)	2011	China (Taiwan)	WPRO	Cohort study	Schizophrenia: 46/3,150 No Schizophrenia: 6,876/613,918	6	2005	NR	>18	Schizophrenia: ICD-9 Diabetes: ICD-9	Age, gender, insurance amount, region, and urbanicity	NOS scores 8
Bresee et al. (59)	2011	Canada	AMRO	Cross-sectional study	Schizophrenia: 48/399 No Schizophrenia: 6,363/120,044	NR	2005	Schizophrenia: 62.1 No Schizophrenia: 49.0	>18	Schizophrenia: Self-report Diabetes: A health professor	Age, sex, income, education, physical activity, smoking status, cardiovascular disease, and total number of chronic medical conditions	AHRQ scores 9
Okumura et al. (60)	2010	Japan	WPRO	Cohort study	Schizophrenia: 333/3,894 No Schizophrenia: 424/4,296	1 Average	2005	Schizophrenia: 49.5 No Schizophrenia: 40.6	Schizophrenia: 45.3 Population control: 44.5	Schizophrenia: DSM IV Diabetes: Discharge diagnosis, hypoglycemic prescription, general practitioner’s diagnosis and treatment	Age, gender	NOS scores 7
Bresee et al. (61)	2010	Canada	AMRO	Cohort study	Schizophrenia: 2,952/28,755 No Schizophrenia: 126,817/2,281,636	10	2006	Schizophrenia: 50.8 No Schizophrenia: 49.5	Schizophrenia: 47.6 No schizophrenia: 45.3	Schizophrenia: ICD-9, ICD-10 Diabetes: the National Diabetes Surveillan System (NDSS)	Age, gender, socioeconomic status, and GP visits	NOS scores 7
Goff et al. (63)	2005	USA	AMRO	Cross-sectional study	Schizophrenia: 87/689 No Schizophrenia: 20/687	NR	2004	Schizophrenia: 73.9 No Schizophrenia: 73.9	Schizophrenia: 40.4 No schizophrenia: 40.4	Schizophrenia: SCID Diabetes: ADA criteria	Age, race and gender	AHRQ scores 8
Hung et al. (62)	2005	China (Taiwan)	WPRO	Cross-sectional study	Schizophrenia: 24/246 No Schizophrenia: 120/1534	NR	NR	Schizophrenia: 55.3 No Schizophrenia: 73.9	Schizophrenia: 37.3 No schizophrenia: NR	Schizophrenia: DSM IV Diabetes: ADA criteria	NR	AHRQ scores 7
Sokal et al. (64)	2004	USA	AMRO	Cross-sectional study	Schizophrenia: 10/97 No Schizophrenia: 167/2,861	NR	NR	Schizophrenia: 63.0 No Schizophrenia: NR	Schizophrenia: 42.4 No schizophrenia: NR	Schizophrenia: NR Diabetes: NR	BMI	AHRQ scores 6
Curkendall et al. (65)	2004	Canada	AMRO	Cohort study	Schizophrenia: 277/3,022 No Schizophrenia: 610/12,088	3	1995	Schizophrenia: 49.5 No Schizophrenia: 49.5	Schizophrenia: 49.6 No schizophrenia: 49.6	Schizophrenia: ICD-9 Diabetes: ICD-9	Gender, age and medical risk factors	NOS scores 7

NR, Not Reported.

### 3.3 Quality assessment

Following the assessment based on the NOS for cohort and case-control studies and the AHRQ criteria for cross-sectional studies, the average NOS score for all included cohort and case-control studies was 7.12. Similarly, the average AHRQ score for cross-sectional studies was 6.73. These scores collectively affirm the high quality of all observational studies incorporated in this meta-analysis. **Table 1** presents the individual scores of each included study, providing a comprehensive overview of the meticulous quality assessment conducted according to the specified criteria. The consistently high scores across these studies underscore the robustness and reliability of the evidence synthesized in this meta-analysis.

### 3.4 Schizophrenia and risk of T2DM

**Figure 2:**
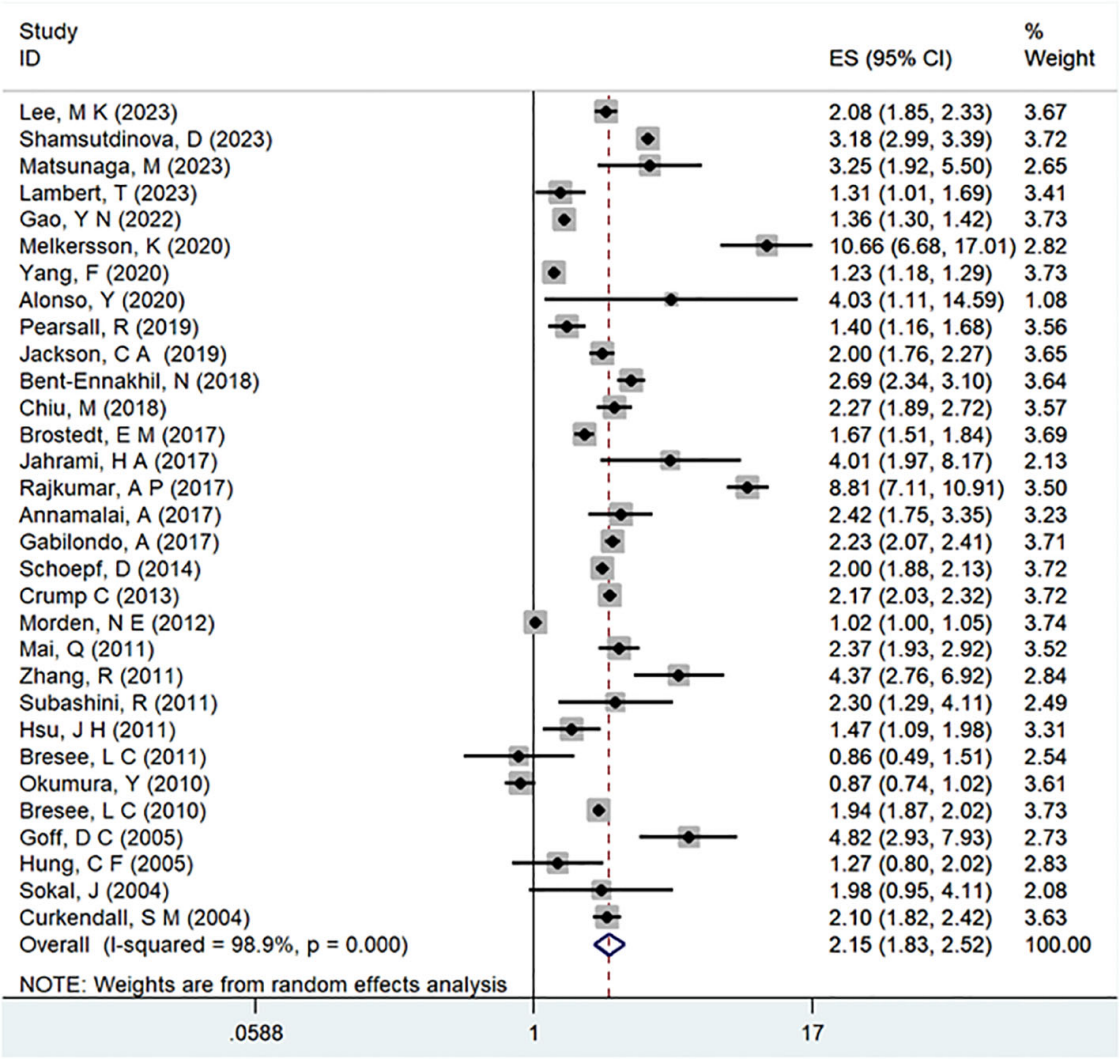
Meta-analysis of the risk of T2DM caused by schizophrenia.

**Figure 3:**
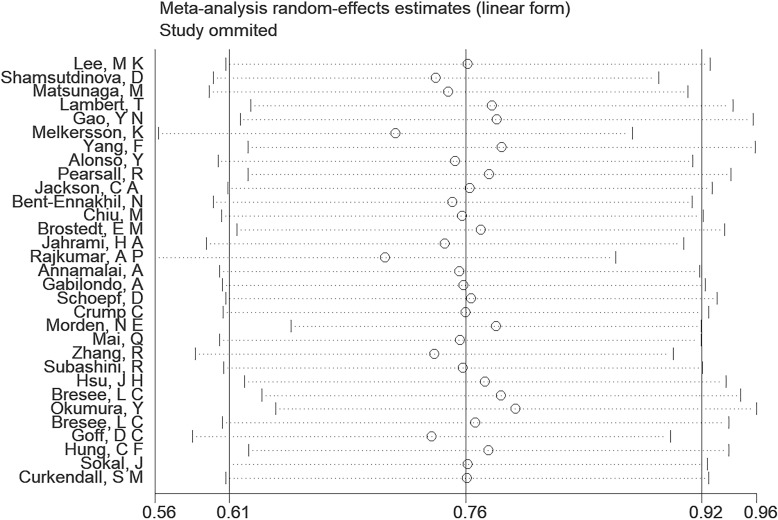
Sensitivity analysis.

A comprehensive analysis of thirty-one observational studies (34–43, 45–65) investigated the relationship between a history of schizophrenia and the risk of T2DM. The pooled results revealed a significant association, indicating that individuals with a history of schizophrenia face a heightened risk of developing T2DM (OR = 2.15; 95% CI: 1.83–2.52; I2 = 98.9%, P < 0.001; **Figure 2**). The substantial heterogeneity, reflected in the I2 statistic, underscores the variability among the included studies, while the low p-value highlights the statistical significance of the observed association. To ensure the robustness of these findings, a sensitivity analysis was conducted. Encouragingly, none of the individual studies within the pool reversed the overall effect size, confirming the stability and reliability of the results (**Figure 3**). These insights contribute valuable knowledge to understanding the link between schizophrenia and the increased risk of T2DM, offering potential implications for clinical practice and avenues for further research.

### 3.5 Subgroup analysis

**Figure 4:**
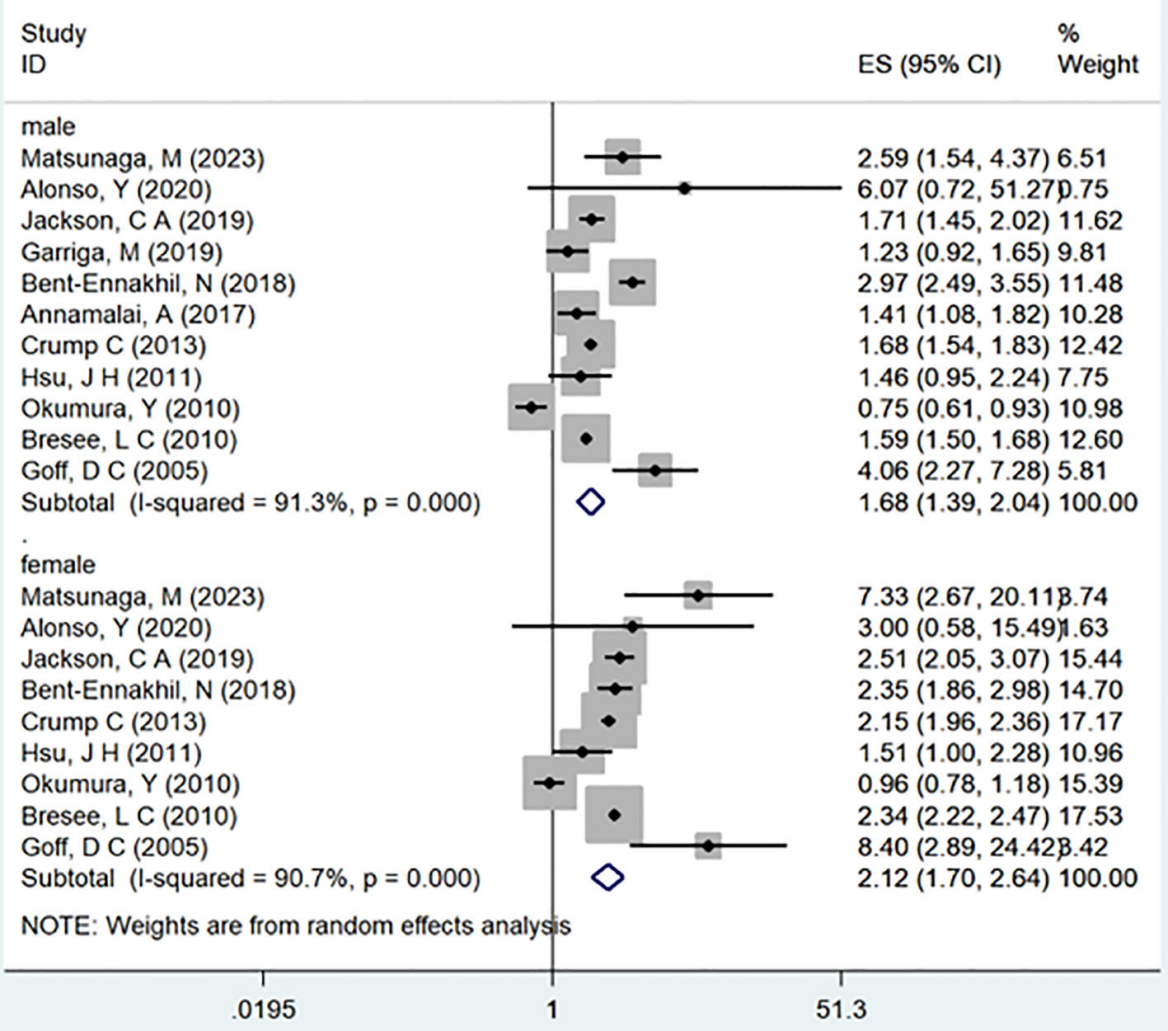
Subgroup for gender.

**Figure 5:**
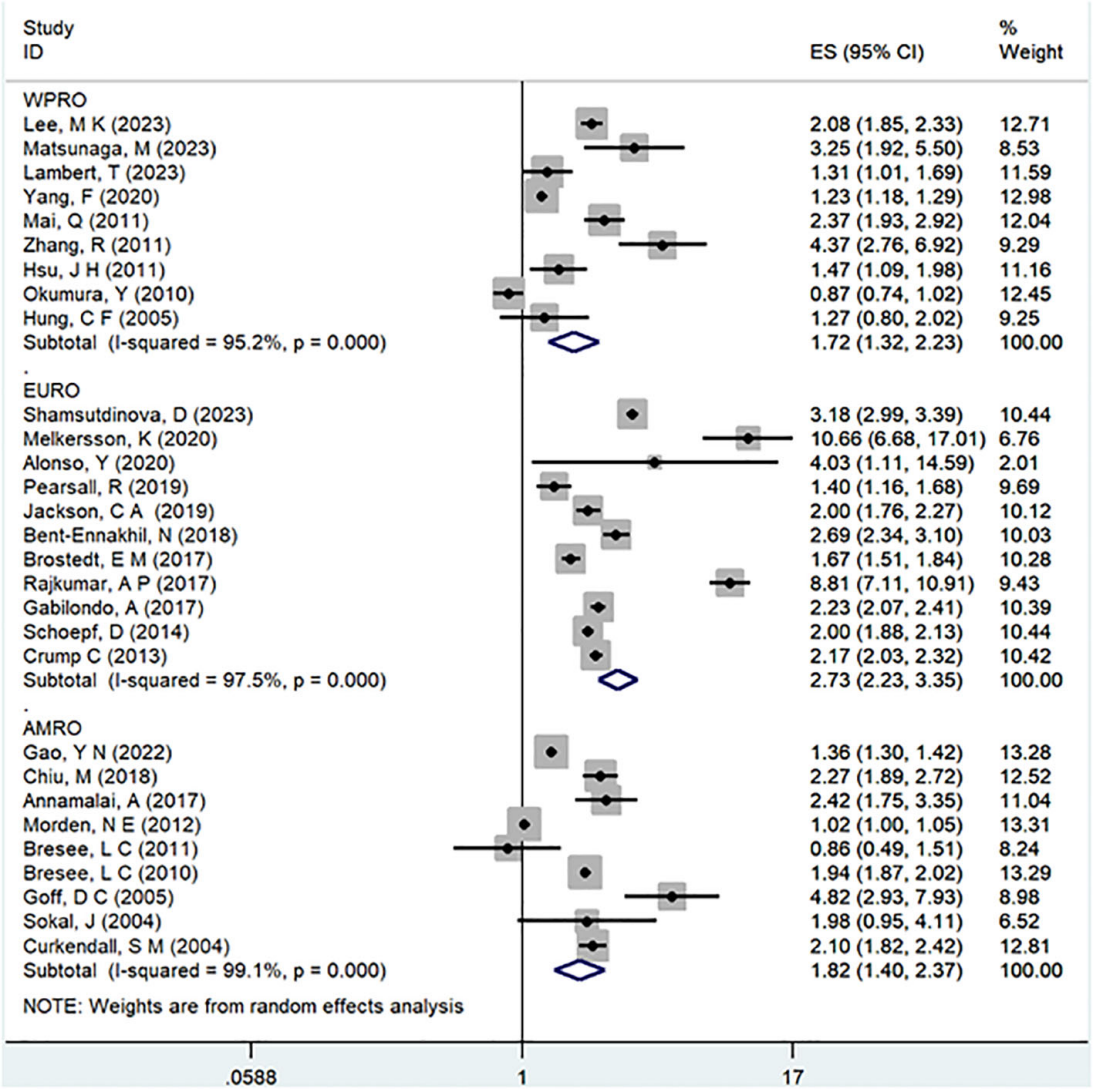
Subgroup for WHO region.

**Figure 6:**
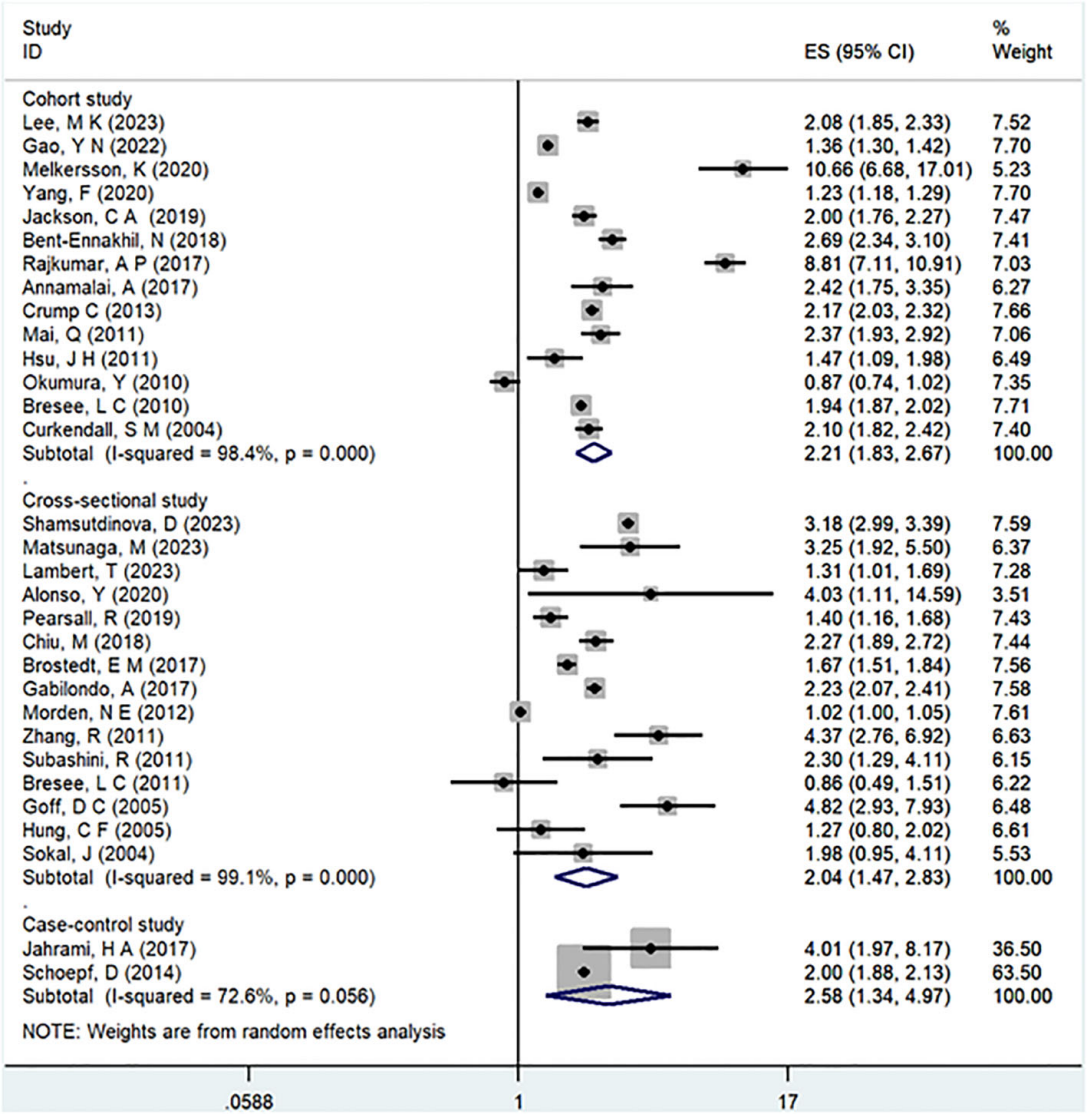
Subgroup for study type.

**Figure 7:**
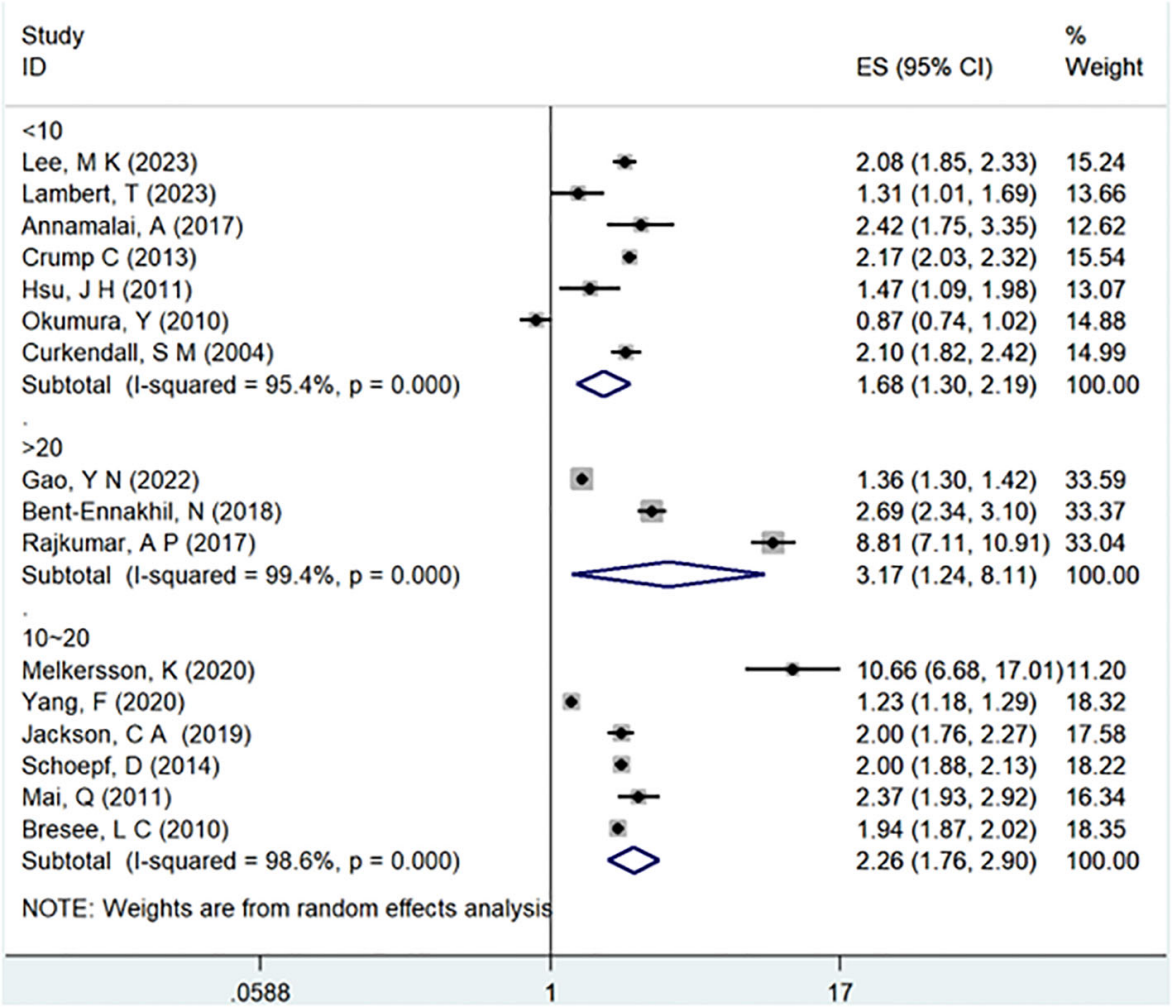
Subgroup for follow-up years.

In the examination of the included studies, a thorough subgroup analysis was conducted based on gender, study type, WHO region, and follow-up time, with detailed results presented in **Table 2**. For gender (**Figure 4**), an in-depth subgroup analysis was performed on eleven studies (35, 41, 43, 44, 46, 51, 53, 58, 60, 61, 63) within the trial comparisons. The findings indicated that females (OR=2.12; 95% CI: 1.70-2.64; I2 = 90.7%, P < 0.001) with a history of schizophrenia face a significantly higher risk of T2DM compared to males (OR=1.68; 95% CI: 1.39-2.04; I2 = 91.3%, P < 0.001). In terms of WHO region (**Figure 5**), a detailed subgroup analysis was conducted on twenty-nine studies (34–43, 45–47, 49–55, 57–65) within the trial comparisons. The studies were categorized into three subgroups: WPRO (35–37, 39, 55, 57, 58, 60, 62), EURO (34, 40–43, 46, 47, 49, 50, 52, 53), and AMRO (38, 45, 51, 54, 59, 61, 63–65). The within-trial comparisons revealed that EURO (OR=2.73; 95% CI: 2.23-3.35; I2 = 97.5%, P < 0.001) had a significantly higher risk of T2DM than WPRO (OR=1.72; 95% CI: 1.32-2.23; I2 = 95.2%, P < 0.001) and AMRO (OR=1.82; 95% CI: 1.40-2.37; I2 = 99.1%, P < 0.001). In the analysis of study types (**Figure 6**), we conducted a subgroup analysis involving thirty-one studies (34–43, 45–65) within the trial comparisons. Among these, fifteen studies (34, 35, 37, 41, 42, 45, 49, 50, 54–56, 59, 62–64) belonged to cross-sectional studies, while two studies (48, 52) were categorized as case-control studies. The remaining fourteen studies fell under the cohort studies category. Across all study types in within-trial comparisons (**Figure 7**), it was consistently observed that schizophrenia poses a significant risk for T2DM. The results for each study type were as follows: cohort study (OR=2.11; 95% CI: 1.83-2.67; I2 = 98.4%, P < 0.001), case-control study (OR=2.58; 95% CI: 1.34-4.97; I2 = 72.6%, P = 0.056), and cross-sectional study (OR=2.04; 95% CI: 1.47-2.83; I2 = 99.1%, P < 0.001). Regarding follow-up years, we conducted a subgroup analysis involving sixteen studies (36–40, 43, 46, 47, 51–53, 57, 58, 60, 61, 65) within the trial comparisons. These studies were further divided into three subgroups based on follow-up duration: <10 years (36, 37, 51, 53, 58, 60, 65), 10-20 years (38, 46, 47), and >20 years (39, 40, 43, 52, 57, 61). The risk of developing T2DM in patients with schizophrenia was found to be associated with the duration of the disease. Notably, the >20 years subgroup showed a significantly higher risk of T2DM (OR=3.17; 95% CI: 1.24-8.11; I2 = 99.4%, P < 0.001) compared to the 10-20 years group (OR=2.26; 95% CI: 1.76-2.90; I2 = 98.6%, P < 0.001) and the <10 years group (OR=1.68; 95% CI: 1.30-2.19; I2 = 95.4%, P < 0.001).

**Table 2:** Subgroup analysis for the risk of T2DM in patients with schizophrenia.

Subgroup	Included studies	OR (95% CI)	Heterogeneity	
			I^2^ (%)	P-values
Gender				
Male	11	1.68(1.39-2.04)	91.3%	0.000
Female	9	2.12(1.70-2.64)	90.7%	0.000
WHO region				
WPRO	9	1.72(1.32-2.23)	95.2	0.000
EURO	11	2.73(2.23-3.35)	97.5	0.000
AMRO	9	1.82(1.40-2.37)	99.1	0.000
Study type				
Cohort study	14	2.11(1.83-2.67)	98.4%	0.000
Cross-sectional study	15	2.04(1.47-2.83)	99.1%	0.000
Case-control study	2	2.58(1.34-4.97)	72.6%	0.056
Follow-up years				
<10	7	1.68(1.30-2.19)	95.4%	0.000
10-20	6	2.26(1.76-2.90)	98.6%	0.000
>20	3	3.17(1.24-8.11)	99.4%	0.000

### 3.6 Publication bias

**Figure 8:**
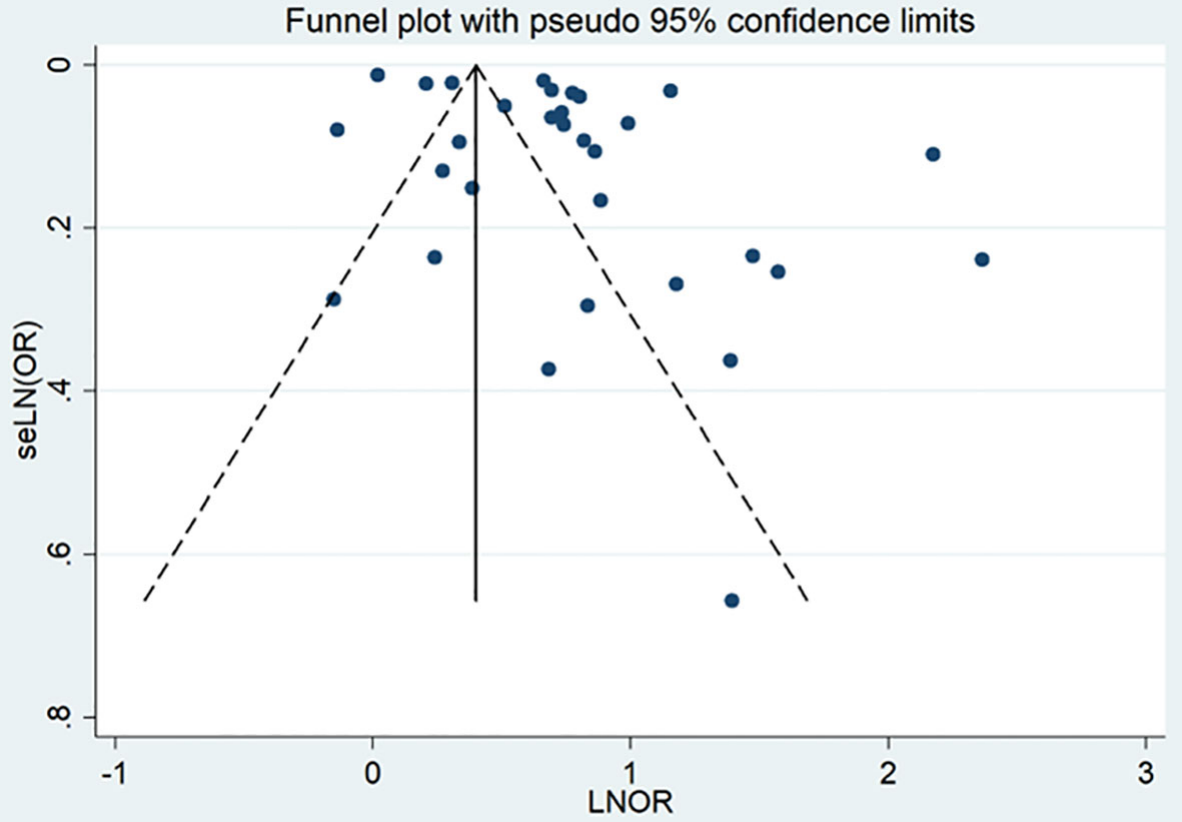
Publication bias of the risk of T2DM caused by schizophrenia.

Upon visually examining the funnel plot, there was no discernible evidence suggesting a significant publication bias in the analysis of schizophrenia disorders and their association with the risk of T2DM (**Figure 8**). However, the Egger’s regression test (P = 0.010) indicated a noteworthy presence of publication bias within the scope of our meta-analysis.

## 4 Discussion

### 4.1 Main findings

This meta-analysis encompasses 32 observational studies, involving 2,007,168 individuals with schizophrenia and 35,883,980 without schizophrenia. It offers a thorough assessment of the correlation between schizophrenia and T2DM. Our findings reveal a notable escalation in the risk of T2DM among individuals with schizophrenia, demonstrating an overall 2.15-fold increase in risk compared to controls without schizophrenia. When considering recent observational studies, these results further substantiate schizophrenia as a significant risk factor for the development of T2DM.

### 4.2 Interpretation of finding

Previous meta-analysis investigated the association between schizophrenia and T2DM (28, 29). The results showed that schizophrenia increased the risk of T2DM. However, they did not analyze subgroups for WHO region, gender, study type and follow-up time. We added more recent studies and analyzed the data according to the above subgroups, so as to provide strong evidence for the association between schizophrenia and T2DM, the previous meta-analysis did not show these meaningful conclusions.

To date, there have been limited studies investigating the association between schizophrenia and T2DM. While various mechanisms underlie the comorbidities between schizophrenia and T2DM, a consensus statement is yet to be established. In terms of genetics, schizophrenia and T2DM share numerous overlapping risk loci (12, 66), including but not limited to chromosomes 1p13, 1p36, 1q21–24, 1q25, 2q14, 2q33, and 2q36. Certain gene regions within these loci may play a role in the pathogenesis of T2DM in individuals with schizophrenia. Prior research has identified a reduction in dendritic spine density in the brains of individuals with schizophrenia (67, 68). This reduction is influenced by both Rho GTPase and the Wnt/β-Catenin pathway through distinct mechanisms (69). These pathways contribute to disruptions in insulin biosynthesis, thereby increasing susceptibility to T2DM in individuals with schizophrenia compared to the general population. Regarding inflammatory factors, a meta-analysis reported elevated levels of IL-6, IL-1β, and TNF-α in the blood and cerebrospinal fluid of individuals with schizophrenia (70). The heightened levels of these cytokines may potentially accelerate the progression of insulin resistance (71). The alterations in the immune system and inflammatory components induced by chronic stress are associated with the molecular mechanisms of T2DM in individuals with schizophrenia (72). Concerning oxidative stress, PON1 emerges as a candidate gene implicated in both schizophrenia and T2DM. The enzyme PON1 plays a crucial role in mitigating oxidative stress and exhibits an inverse relationship with cytokine levels (73). In individuals diagnosed with schizophrenia, there is a notable reduction in PON1 enzyme activity, and this diminishing trend adversely impacts the normal functioning of β-cells. Patients with schizophrenia often require prolonged use of antipsychotic medications, including olanzapine, clozapine, haloperidol, sertindole, and other commonly prescribed antipsychotics. These medications are associated with an increased susceptibility to metabolic disorders, particularly disruptions in glucose homeostasis leading to the progression from insulin resistance to T2DM (74, 75). Furthermore, the detrimental lifestyle habits, such as smoking, and cognitive dysfunction exhibited by individuals with schizophrenia can directly or indirectly influence the daily blood sugar levels of these patients (75, 76). This, in turn, contributes to a heightened risk of T2DM incidence.

In the subgroup analysis, several noteworthy results emerged that could provide valuable insights for clinicians. Notably, females with a history of schizophrenia exhibit a significantly elevated risk of developing T2DM when compared to their male counterparts. This finding underscores a significant susceptibility of females to T2DM, aligning with prior research that indicated women taking antipsychotics faced a higher likelihood of T2DM development compared to men (77). This heightened risk in females may be attributed to factors such as weight gain and the emergence of insulin resistance mediated by sex-related genes. Notably, women are more prone to developing T2DM following antipsychotic intervention, a phenomenon associated with increased body weight (78). Furthermore, the expression of specific sex-related genes appears to render women more susceptible to insulin resistance than men (79). A comprehensive meta-analysis of cross-sectional studies revealed intriguing differences between genders in the context of T2DM. In men with T2DM, significantly lower testosterone levels were observed compared to controls, while women exhibited higher testosterone levels. Prospective studies complement these findings, indicating that men with elevated testosterone levels experience a 42 percent reduction in the risk of developing T2DM compared to controls. Conversely, heightened testosterone levels in women seem to correlate with an increased risk of T2DM development (80). In the context of follow-up times subgroup analysis, our calculations align with prior research, supporting the conclusion that the prevalence of T2DM in patients with schizophrenia increases with the duration of the disease (81). This underscores the importance of considering the longitudinal aspect when evaluating the association between schizophrenia and T2DM.

### 4.3 Implications and limitations

Our meta-analysis examining the relationship between a history of schizophrenia and the risk of T2DM reinforces the idea that schizophrenia constitutes a risk factor for the development of T2DM. This underscores the importance of heightened awareness regarding the risk of T2DM in individuals with schizophrenia, potentially aiding early clinicians in the identification of patients at risk for T2DM. Nonetheless, it is essential to acknowledge certain limitations inherent in our study. The data included in our analysis exhibited high heterogeneity, and despite a thorough examination, the source of this heterogeneity remained unidentified. Nevertheless, a majority of the studies incorporated in our analysis meticulously controlled for numerous confounding factors, enhancing the reliability of our conclusions. To further advance the field, future research endeavors should consider incorporating additional subgroups to diversify and enrich the scope of investigation. It is worth noting that our meta-analysis did not include covariate analysis. However, the studies included in our analysis implemented control measures for adjusted confounding factors, contributing to robust confounding bias control. This strengthens the credibility of our study’s findings and facilitates seamless translation into clinical practice.

## 5 Conclusions

This meta-analysis suggests that schizophrenia heightens the risk of developing T2DM. However, a more precise explanation for this phenomenon necessitates further research. The findings from our meta-analysis can prove invaluable in shaping new strategies for the prevention and treatment of schizophrenia.

## Data Availability

The original contributions presented in the study are included in the article. Further inquiries can be directed to the corresponding author.

## References

[1] GBD Organization . Global, regional, and national burden of 12 mental disorders in 204 countries and territories, 1990-2019: a systematic analysis for the Global Burden of Disease Study 2019. Lancet Psychiatry. (2022) 9:137–50. doi: 10.1016/S2215-0366(21)00395-3 PMC8776563 35026139

[2] Correll CU Solmi M Croatto G Schneider LK Rohani-Montez SC Fairley L . Mortality in people with schizophrenia: a systematic review and meta-analysis of relative risk and aggravating or attenuating factors. World Psychiatry. (2022) 21:248–71. doi: 10.1002/wps.20994 PMC9077617 35524619

[3] Vancampfort D Rosenbaum S Schuch F Ward PB Richards J Mugisha J . Cardiorespiratory fitness in severe mental illness: A systematic review and meta-analysis. Sports Med. (2017) 47:343–52. doi: 10.1007/s40279-016-0574-1 27299747

[4] Dragioti E Radua J Solmi M Gosling CJ Oliver D Lascialfari F . Impact of mental disorders on clinical outcomes of physical diseases: an umbrella review assessing population attributable fraction and generalized impact fraction. World Psychiatry. (2023) 22:86–104. doi: 10.1002/wps.21068 36640414 PMC9840513

[5] Laursen TM . Causes of premature mortality in schizophrenia: a review of literature published in 2018. Curr Opin Psychiatry. (2019) 32:388–93. doi: 10.1097/YCO.0000000000000530 31135491

[6] Mitchell AJ Vancampfort D Sweers K van Winkel R Yu W De Hert M . Prevalence of metabolic syndrome and metabolic abnormalities in schizophrenia and related disorders–a systematic review and meta-analysis. Schizophr bulletin. (2013) 39:306–18. doi: 10.1093/schbul/sbr148 PMC3576174 22207632

[7] Key global findings 2021 (2021). Available online at: https://diabetesatlas.org (Accessed 13 December 2023).

[8] Henderson DC Vincenzi B Andrea NV Ulloa M Copeland PM . Pathophysiological mechanisms of increased cardiometabolic risk in people with schizophrenia and other severe mental illnesses. Lancet Psychiatry. (2015) 2:452–64. doi: 10.1016/S2215-0366(15)00115-7 26360288

[9] Lindenmayer JP Czobor P Volavka J Citrome L Sheitman B McEvoy JP . Changes in glucose and cholesterol levels in patients with schizophrenia treated with typical or atypical antipsychotics. Am J Psychiatry. (2003) 160:290–6. doi: 10.1176/appi.ajp.160.2.290 12562575

[10] Perry BI McIntosh G Weich S Singh S Rees K . The association between first-episode psychosis and abnormal glycaemic control: systematic review and meta-analysis. Lancet Psychiatry. (2016) 3:1049–58. doi: 10.1016/S2215-0366(16)30262-0 27720402

[11] Pillinger T Beck K Gobjila C Donocik JG Jauhar S Howes OD . Impaired glucose homeostasis in first-episode schizophrenia: A systematic review and meta-analysis. JAMA Psychiatry. (2017) 74:261–9. doi: 10.1001/jamapsychiatry.2016.3803 PMC6352957 28097367

[12] Rødevand L Rahman Z Hindley GFL Smeland OB Frei O Tekin TF . Characterizing the shared genetic underpinnings of schizophrenia and cardiovascular disease risk factors. Am J Psychiatry. (2023) 180:815–26. doi: 10.1176/appi.ajp.20220660 PMC11780279 37752828

[13] Del Bosque-Plata L Martínez-Martínez E Espinoza-Camacho M Gragnoli C . The role of TCF7L2 in type 2 diabetes. Diabetes. (2021) 70(6):1220–8. doi: 10.2337/db20-0573 PMC8275893 34016596

[14] Hansen T Ingason A Djurovic S Melle I Fenger M Gustafsson O . At-risk variant in TCF7L2 for type II diabetes increases risk of schizophrenia. Biol Psychiatry. (2011) 70:59–63. doi: 10.1016/j.biopsych.2011.01.031 21414605

[15] Martland R Teasdale S Murray RM Gardner-Sood P Smith S Ismail K . Dietary intake, physical activity and sedentary behaviour patterns in a sample with established psychosis and associations with mental health symptomatology. psychol Med. (2023) 53:1565–75. doi: 10.1017/S0033291721003147 PMC10009388 34420532

[16] Singh R Bansal Y Medhi B Kuhad A . Antipsychotics-induced metabolic alterations: Recounting the mechanistic insights, therapeutic targets and pharmacological alternatives. Eur J Pharmacol. (2019) 844:231–40. doi: 10.1016/j.ejphar.2018.12.003 30529195

[17] Raben AT Marshe VS Chintoh A Gorbovskaya I Müller DJ Hahn MK . The complex relationship between antipsychotic-induced weight gain and therapeutic benefits: A systematic review and implications for treatment. Front Neurosci. (2017) 11:741. doi: 10.3389/fnins.2017.00741 29403343 PMC5786866

[18] Carli M Kolachalam S Longoni B Pintaudi A Baldini M Aringhieri S . Atypical antipsychotics and metabolic syndrome: from molecular mechanisms to clinical differences. Pharm (Basel). (2021) 14(3):238. doi: 10.3390/ph14030238 PMC8001502 33800403

[19] Scheen AJ . Metabolic disorders induced by psychotropic drugs. Annales d’endocrinologie. (2023) 84:357–63. doi: 10.1016/j.ando.2023.03.006 36963753

[20] Poulos J Normand ST Zelevinsky K Newcomer JW Agniel D Abing HK . Antipsychotics and the risk of diabetes and death among adults with serious mental illnesses. psychol Med. (2023) 53:7677–84. doi: 10.1017/S0033291723001502 PMC10758338 37753625

[21] Singh R Stogios N Smith E Lee J Maksyutynsk K Au E . Gut microbiome in schizophrenia and antipsychotic-induced metabolic alterations: a scoping review. Ther Adv psychopharmacology. (2022) 12:20451253221096525. doi: 10.1177/20451253221096525 PMC9118432 35600753

[22] Jin DM Morton JT Bonneau R . Meta-analysis of the human gut microbiome uncovers shared and distinct microbial signatures between diseases. mSystems. (2024) 9(8):e0029524. doi: 10.1128/msystems.00295-24 39078158 PMC11334437

[23] Multimorbidity: clinical assessment and management: NICE guideline [NG56] (2016). Available online at: https://www.nice.org.uk/guidance/NG56 (Accessed 13 December 2023).

[24] Hagi K Nosaka T Dickinson D Lindenmayer JP Lee J Friedman J . Association between cardiovascular risk factors and cognitive impairment in people with schizophrenia: A systematic review and meta-analysis. JAMA Psychiatry. (2021) 78:510–8. doi: 10.1001/jamapsychiatry.2021.0015 PMC7931134 33656533

[25] Scheuer SH Kosjerina V Lindekilde N Pouwer F Carstensen B Jørgensen ME . Severe mental illness and the risk of diabetes complications: A nationwide, register-based cohort study. J Clin Endocrinol Metab. (2022) 107:e3504–e14. doi: 10.1210/clinem/dgac204 35359003

[26] Ali S Santomauro D Ferrari AJ Charlson F . Schizophrenia as a risk factor for cardiovascular and metabolic health outcomes: a comparative risk assessment. Epidemiol Psychiatr Sci. (2023) 32:e8. doi: 10.1017/S2045796023000045 36756905 PMC9971851

[27] Ivers NM Jiang M Alloo J Singer A Ngui D Casey CG . Diabetes Canada 2018 clinical practice guidelines: Key messages for family physicians caring for patients living with type 2 diabetes. Can Fam Physician. (2018) 65(1):14–24. PMC6347316 30674509

[28] Stubbs B Vancampfort D De Hert M Mitchell AJ . The prevalence and predictors of type two diabetes mellitus in people with schizophrenia: a systematic review and comparative meta-analysis. Acta psychiatrica Scandinavica. (2015) 132:144–57. doi: 10.1111/acps.12439 25943829

[29] Vancampfort D Correll CU Galling B Probst M De Hert M Ward PB . Diabetes mellitus in people with schizophrenia, bipolar disorder and major depressive disorder: a systematic review and large scale meta-analysis. World Psychiatry. (2016) 15:166–74. doi: 10.1002/wps.20309 PMC4911762 27265707

[30] Page MJ McKenzie JE Bossuyt PM Boutron I Hoffmann TC Mulrow CD . The PRISMA 2020 statement: an updated guideline for reporting systematic reviews. BMJ. (2021). doi: 10.1136/bmj.n71 PMC8005924 33782057

[31] Taylor KS Mahtani KR Aronson JK . Summarising good practice guidelines for data extraction for systematic reviews and meta-analysis. BMJ Evid Based Med. (2021) 26:88–90. doi: 10.1136/bmjebm-2020-111651 33632720

[32] Wells GA Wells G Shea B Shea B O’Connell D Peterson J . The Newcastle-Ottawa Scale (NOS) for Assessing the Quality of Nonrandomised Studies in Meta-Analyses2014. (2014). Available online at: https://www.semanticscholar.org/paper/The-Newcastle-Ottawa-Scale-(NOS)-for-Assessing-the-Wells-Wells/c293fb316b6176154c3fdbb8340a107d9c8c82bf.

[33] Clair JS . A new model of tracheostomy care: closing the research–practice gap. In: Henriksen K Battles JB Marks ES Lewin DI , editors Advances in patient safety: from research to implementation. Vol. 3. Implementation issues. Rockville (MD): Agency for Healthcare Research and Quality (US) (2005); 2005 Feb. Table 1, AHRQ scale of research grades and levels. (Available from: https://www.ncbi.nlm.nih.gov/books/NBK20542/table/A5857/).

[34] Shamsutdinova D Das-Munshi J Ashworth M Roberts A Stahl D . Predicting type 2 diabetes prevalence for people with severe mental illness in a multi-ethnic East London population. Int J Med informatics. (2023) 172:105019. doi: 10.1016/j.ijmedinf.2023.105019 36787689

[35] Matsunaga M Li Y He Y Kishi T Tanihara S Iwata N . Physical, psychiatric, and social comorbidities of individuals with schizophrenia living in the community in Japan. Int J Environ Res Public Health. (2023) 20(5):4336. doi: 10.3390/ijerph20054336 PMC10001945 36901345

[36] Lee MK Lee SY Sohn SY Ahn J Han K Lee JH . Type 2 diabetes and its association with psychiatric disorders in young adults in South Korea. JAMA network Open. (2023) 6:e2319132. doi: 10.1001/jamanetworkopen.2023.19132 37389877 PMC10314316

[37] Lambert T Middleton T Chen R Sureshkumar P . Prevalence of, and factors associated with, diabetes mellitus in people with severe mental illness attending a multidisciplinary, outpatient cardiometabolic health assessment service. BMJ Open Diabetes Res Care. (2023) 11(1). doi: 10.1136/bmjdrc-2022-003055 PMC9853146 36653062

[38] Gao YN Olfson M . National trends in metabolic risk of psychiatric inpatients in the United States during the atypical antipsychotic era. Schizophr Res. (2022) 248:320–8. doi: 10.1016/j.schres.2022.09.023 PMC10135373 36155305

[39] Yang F Ma Q Liu J Ma B Guo M Liu F . Prevalence and major risk factors of type 2 diabetes mellitus among adult psychiatric inpatients from 2005 to 2018 in Beijing, China: a longitudinal observational study. BMJ Open Diabetes Res Care. (2020) 8(1). doi: 10.1136/bmjdrc-2019-000996 PMC7059541 32139600

[40] Melkersson K . Schizophrenia- or schizoaffective disorder diagnosis and the risk for subsequent type 1- or type 2 diabetes mellitus: a Swedish nationwide register-based cohort study. Neuro Endocrinol letters. (2020) 41:245–54. 33315340

[41] Alonso Y Valiente-Pallejà A Verge B Vilella E Martorell L . High frequency of clinical conditions commonly associated with mitochondrial disorders in schizophrenia. Acta neuropsychiatrica. (2020) 32:265–9. doi: 10.1017/neu.2020.16 32329429

[42] Pearsall R Shaw RJ McLean G Connolly M Hughes KA Boyle JG . Health screening, cardiometabolic disease and adverse health outcomes in individuals with severe mental illness. BJPsych Open. (2019) 5:e97. doi: 10.1192/bjo.2019.76 31699180 PMC6854356

[43] Jackson CA Fleetwood K Kerssens J Smith DJ Mercer S Wild SH . Incidence of type 2 diabetes in people with a history of hospitalization for major mental illness in scotland, 2001-2015: A retrospective cohort study. Diabetes Care. (2019) 42:1879–85. doi: 10.2337/dc18-2152 31471379

[44] Garriga M Wium-Andersen MK Wium-Andersen IK Nordentoft M Osler M . Birth dimensions, severe mental illness and risk of type 2 diabetes in a cohort of Danish men born in 1953. Eur psychiatry: J Assoc Eur Psychiatrists. (2019) 62:1–9. doi: 10.1016/j.eurpsy.2019.08.015 31505317

[45] Chiu M Rahman F Vigod S Wilton AS Kurdyak P . Temporal trends in cardiovascular disease risk factor profiles in a population-based schizophrenia sample: a repeat cross-sectional study. J Epidemiol Community Health. (2018) 72:71–7. doi: 10.1136/jech-2017-209565 29061843

[46] Bent-Ennakhil N Cécile Périer M Sobocki P Gothefors D Johansson G Milea D . Incidence of cardiovascular diseases and type-2-diabetes mellitus in patients with psychiatric disorders. Nordic J Psychiatry. (2018) 72:455–61. doi: 10.1080/08039488.2018.1463392 30513230

[47] Rajkumar AP Horsdal HT Wimberley T Cohen D Mors O Børglum AD . Endogenous and antipsychotic-related risks for diabetes mellitus in young people with schizophrenia: A danish population-based cohort study. Am J Psychiatry. (2017) 174:686–94. doi: 10.1176/appi.ajp.2016.16040442 28103712

[48] Jahrami HA Faris MAE Saif ZQ Hammad LH . Assessing dietary and lifestyle risk factors and their associations with disease comorbidities among patients with schizophrenia: A case-control study from Bahrain. Asian J Psychiatry. (2017) 28:115–23. doi: 10.1016/j.ajp.2017.03.036 28784363

[49] Gabilondo A Alonso-Moran E Nuño-Solinis R Orueta JF Iruin A . Comorbidities with chronic physical conditions and gender profiles of illness in schizophrenia. Results from PREST, a new health dataset. J psychosomatic Res. (2017) 93:102–9. doi: 10.1016/j.jpsychores.2016.12.011 28107885

[50] Brostedt EM Msghina M Persson M Wettermark B . Health care use, drug treatment and comorbidity in patients with schizophrenia or non-affective psychosis in Sweden: a cross-sectional study. BMC Psychiatry. (2017) 17:416. doi: 10.1186/s12888-017-1582-x 29284436 PMC5747108

[51] Annamalai A Kosir U Tek C . Prevalence of obesity and diabetes in patients with schizophrenia. World J Diabetes. (2017) 8:390–6. doi: 10.4239/wjd.v8.i8.390 PMC5561038 28861176

[52] Schoepf D Uppal H Potluri R Heun R . Physical comorbidity and its relevance on mortality in schizophrenia: a naturalistic 12-year follow-up in general hospital admissions. Eur Arch Psychiatry Clin Neurosci. (2014) 264:3–28. doi: 10.1007/s00406-013-0436-x 23942824

[53] Crump C Winkleby MA Sundquist K Sundquist J . Comorbidities and mortality in persons with schizophrenia: a Swedish national cohort study. Am J Psychiatry. (2013) 170:324–33. doi: 10.1176/appi.ajp.2012.12050599 23318474

[54] Morden NE Lai Z Goodrich DE MacKenzie T McCarthy JF Austin K . Eight-year trends of cardiometabolic morbidity and mortality in patients with schizophrenia. Gen Hosp Psychiatry. (2012) 34:368–79. doi: 10.1016/j.genhosppsych.2012.02.009 PMC3383866 22516216

[55] Zhang R Hao W Pan M Wang C Zhang X Chen DC . The prevalence and clinical-demographic correlates of diabetes mellitus in chronic schizophrenic patients receiving clozapine. Hum psychopharmacology. (2011) 26:392–6. doi: 10.1002/hup.1220 21826737

[56] Subashini R Deepa M Padmavati R Thara R Mohan V . Prevalence of diabetes, obesity, and metabolic syndrome in subjects with and without schizophrenia (CURES-104). J postgraduate Med. (2011) 57:272–7. doi: 10.4103/0022-3859.90075 22120854

[57] Mai Q Holman CD Sanfilippo FM Emery JD Preen DB . Mental illness related disparities in diabetes prevalence, quality of care and outcomes: a population-based longitudinal study. BMC Med. (2011) 9:118. doi: 10.1186/1741-7015-9-118 22044777 PMC3215928

[58] Hsu JH Chien IC Lin CH Chou YJ Chou P . Incidence of diabetes in patients with schizophrenia: a population-based study. Can J Psychiatry Rev Can psychiatrie. (2011) 56:19–26. doi: 10.1177/070674371105600105 21324239

[59] Bresee LC Majumdar SR Patten SB Johnson JA . Diabetes, cardiovascular disease, and health care use in people with and without schizophrenia. Eur psychiatry: J Assoc Eur Psychiatrists. (2011) 26:327–32. doi: 10.1016/j.eurpsy.2010.05.003 20634043

[60] Okumura Y Ito H Kobayashi M Mayahara K Matsumoto Y Hirakawa J . Prevalence of diabetes and antipsychotic prescription patterns in patients with schizophrenia: a nationwide retrospective cohort study. Schizophr Res. (2010) 119:145–52. doi: 10.1016/j.schres.2010.02.1061 20304611

[61] Bresee LC Majumdar SR Patten SB Johnson JA . Prevalence of cardiovascular risk factors and disease in people with schizophrenia: a population-based study. Schizophr Res. (2010) 117:75–82. doi: 10.1016/j.schres.2009.12.016 20080392

[62] Hung CF Wu CK Lin PY . Diabetes mellitus in patients with schizophrenia in Taiwan. Prog Neuropsychopharmacol Biol Psychiatry. (2005) 29:523–7. doi: 10.1016/j.pnpbp.2005.01.003 15866353

[63] Goff DC Sullivan LM McEvoy JP Meyer JM Nasrallah HA Daumit GL . A comparison of ten-year cardiac risk estimates in schizophrenia patients from the CATIE study and matched controls. Schizophr Res. (2005) 80:45–53. doi: 10.1016/j.schres.2005.08.010 16198088

[64] Sokal J Messias E Dickerson FB Kreyenbuhl J Brown CH Goldberg RW . Comorbidity of medical illnesses among adults with serious mental illness who are receiving community psychiatric services. J nervous Ment disease. (2004) 192:421–7. doi: 10.1097/01.nmd.0000130135.78017.96 15167405

[65] Curkendall SM Mo J Glasser DB Rose Stang M Jones JK . Cardiovascular disease in patients with schizophrenia in Saskatchewan, Canada. J Clin Psychiatry. (2004) 65:715–20. doi: 10.4088/JCP.v65n0519 15163261

[66] Lin PI Shuldiner AR . Rethinking the genetic basis for comorbidity of schizophrenia and type 2 diabetes. Schizophr Res. (2010) 123:234–43. doi: 10.1016/j.schres.2010.08.022 20832248

[67] Glantz LA Lewis DA . Decreased dendritic spine density on prefrontal cortical pyramidal neurons in schizophrenia. Arch Gen Psychiatry. (2000) 57:65–73. doi: 10.1001/archpsyc.57.1.65 10632234

[68] Konopaske GT Lange N Coyle JT Benes FM . Prefrontal cortical dendritic spine pathology in schizophrenia and bipolar disorder. JAMA Psychiatry. (2014) 71:1323–31. doi: 10.1001/jamapsychiatry.2014.1582 PMC5510541 25271938

[69] Mizuki Y Sakamoto S Okahisa Y Yada Y Hashimoto N Takaki M . Mechanisms underlying the comorbidity of schizophrenia and type 2 diabetes mellitus. Int J Neuropsychopharmacol. (2021) 24:367–82. doi: 10.1093/ijnp/pyaa097 PMC8130204 33315097

[70] Capuzzi E Bartoli F Crocamo C Clerici M Carrà G . Acute variations of cytokine levels after antipsychotic treatment in drug-naïve subjects with a first-episode psychosis: A meta-analysis. Neurosci Biobehav Rev. (2017) 77:122–8. doi: 10.1016/j.neubiorev.2017.03.003 28285148

[71] Reinehr T . Inflammatory markers in children and adolescents with type 2 diabetes mellitus. Clinica chimica acta; Int J Clin Chem. (2019) 496:100–7. doi: 10.1016/j.cca.2019.07.006 31276632

[72] van Beveren NJ Schwarz E Noll R Guest PC Meijer C de Haan L . Evidence for disturbed insulin and growth hormone signaling as potential risk factors in the development of schizophrenia. Trans Psychiatry. (2014) 4:e430. doi: 10.1038/tp.2014.52 PMC4150237 25158005

[73] Moheimani RS Bhetraratana M Yin F Peters KM Gornbein J Araujo JA . Increased cardiac sympathetic activity and oxidative stress in habitual electronic cigarette users: implications for cardiovascular risk. JAMA Cardiol. (2017) 2:278–84. doi: 10.1001/jamacardio.2016.5303 PMC5626008 28146259

[74] Burschinski A Schneider-Thoma J Chiocchia V Schestag K Wang D Siafis S . Metabolic side effects in persons with schizophrenia during mid- to long-term treatment with antipsychotics: a network meta-analysis of randomized controlled trials. World Psychiatry. (2023) 22:116–28. doi: 10.1002/wps.21036 PMC9840505 36640396

[75] Leucht S Schneider-Thoma J Burschinski A Peter N Wang D Dong S . Long-term efficacy of antipsychotic drugs in initially acutely ill adults with schizophrenia: systematic review and network meta-analysis. World Psychiatry. (2023) 22:315–24. doi: 10.1002/wps.21089 PMC10168166 37159349

[76] Ward M Druss B . The epidemiology of diabetes in psychotic disorders. Lancet Psychiatry. (2015) 2:431–51. doi: 10.1016/S2215-0366(15)00007-3 26360287

[77] Seeman MV . Secondary effects of antipsychotics: women at greater risk than men. Schizophr bulletin. (2009) 35:937–48. doi: 10.1093/schbul/sbn023 PMC2728808 18400811

[78] Basu A Meltzer HY . Differential trends in prevalence of diabetes and unrelated general medical illness for schizophrenia patients before and after the atypical antipsychotic era. Schizophr Res. (2006) 86:99–109. doi: 10.1016/j.schres.2006.04.014 16753284

[79] Mittendorfer B . Insulin resistance: sex matters. Curr Opin Clin Nutr Metab Care. (2005) 8:367–72. doi: 10.1097/01.mco.0000172574.64019.98 15930959

[80] Ding EL Song Y Malik VS Liu S . Sex differences of endogenous sex hormones and risk of type 2 diabetes: a systematic review and meta-analysis. Jama. (2006) 295:1288–99. doi: 10.1001/jama.295.11.1288 16537739

[81] Philippe A Vaiva G Casadebaig F . Data on diabetes from the French cohort study in schizophrenia. Eur psychiatry: J Assoc Eur Psychiatrists. (2005) 20 Suppl 4:S340–4. doi: 10.1016/S0924-9338(05)80188-9 16459248

